# The Sex Specific Association Between Maternal Gestational Diabetes and Offspring Metabolic Status at 1 Year of Age

**DOI:** 10.3389/fendo.2020.608125

**Published:** 2021-02-09

**Authors:** Qinwen Du, Yishai Sompolinsky, Asnat Walfisch, Huiping Zhong, Yan Liu, Weiwei Feng

**Affiliations:** ^1^ Department of Gynecology and Obstetrics, Ruijin Hospital, Shanghai Jiaotong University School of Medicine, Shanghai, China; ^2^ Department of Obstetrics and Gynecology, Hadassah Mt. Scopus, The Hebrew University, Jerusalem, Israel

**Keywords:** in utero hyperglycemia, GDM, childhoodobesity, early postnatal period, developmental origins of health, developmental origins of adult health and disease

## Abstract

Previous studies showed the association between maternal GDM and long-term effects of overweight in offspring. However, the nature of this association in the early postnatal period is still undetermined. The aim of this prospective cohort study was to evaluate whether maternal GDM is associated with overweight and obesity status in offspring at age 1 year. We studied 1167 infants born at a large obstetrical care hospital including 778 normal glucose tolerance (NGT) and 389 GDM pregnancies, matched in a 1:2 ratio according to offspring’s gender, during the years 2016–2017. Overweight and obesity status in offspring of both groups were evaluated at 1 year of age through questionnaires. Infant outcomes were defined according to the WHO Child Growth Standards based on the length-based BMI-for-age. Female offspring from the GDM group exhibited a higher mean BMI (17.2 vs. 16.6, *p* < 0.01), a higher rate of obesity (13.9% vs. 7.7%; *p* < 0.05), and overweight (33.1% vs. 23.5%; *p* < 0.05) as compared to the NGT female group. In the multivariable regression model, maternal GDM was found to be independently and significantly associated with overweight or obesity in 1-year aged female offspring only (OR 1.61, 95% CI 1.09–2.37, *p* < 0.05). We found a sex specific association between maternal GDM and the overweight risk only in female offspring at 1 year of age.

## Introduction

Gestational diabetes mellitus (GDM) is a common complication during pregnancy, affecting approximately overall 5%-15% of women, and 6.5–14.8% in Han Chinese population (1–4). As suggested by David Barker and later repeatedly shown by others, the intrauterine life, as well as the early postnatal period, are of critical significance in shaping later metabolic health (5). Previous studies indicated that GDM may increase childhood obesity in offspring (6–9). In turn, overweight and obesity during childhood are later associated with an increased risk for multiple metabolic morbidities during adulthood (10). Moreover, a recent study showed that acceleration of BMI in early childhood is related to the risk of sustained obesity (11) and to a higher incidence of “metabolic syndrome” and type 2 diabetes mellitus (12). Obesity increases risk of many adverse outcomes, however, the relationship between GDM and the early origins of obesity is still unclear.

The association between in utero hyperglycemia and fetal metabolic change was initially described in the Native American Pima population (13). In utero hyperglycemia exposure was shown to increase the risk of obesity in offspring from 5 years of age. Previous study showed that this association remained significant after adjusted for confounding effects of lifestyle characteristics and genetic variation (14). However, it is still unknown that the similar programming effects occur in maternal hyperglycemia in the early postnatal period. Previous studies that examined the risk association between maternal GDM and childhood obesity in the offspring were limited by their designs, differences in the definitions of maternal hyperglycemia and GDM, and in adjustments for confounding factors (15, 16). Inconsistent results were also published (17–19).

The aim of this study was to ascertain the independent effect of maternal GDM on childhood obesity in offspring during the first year of life.

## Materials and Methods

### Study Population

This study was a prospective cohort study of 1444 Chinese Han mother-child pairs were identified. The women gave birth in a large obstetrical care center located in Ruijin hospital, Shanghai, China between January 2016 and December 2017.

The inclusion criteria were: 1) Singleton pregnancy; 2) Chinese Han origin; 3) Completion of a 2 h, 75-g oral glucose tolerance test (OGTT) at 24–28 gestational weeks; 4) No history of a pre-pregnancy or early pregnancy abnormal glucose tolerance; 5) Maternal age between 18 and 45 years; 7) Live infant with no known congenital malformations, and 9) Access to complete medical history.

A total of 277 women were excluded from the study: 118 women did not agree to participate and 159 women could not provide key data regarding the infant’s metabolic status at 1 year of age.

**Figure 1:**
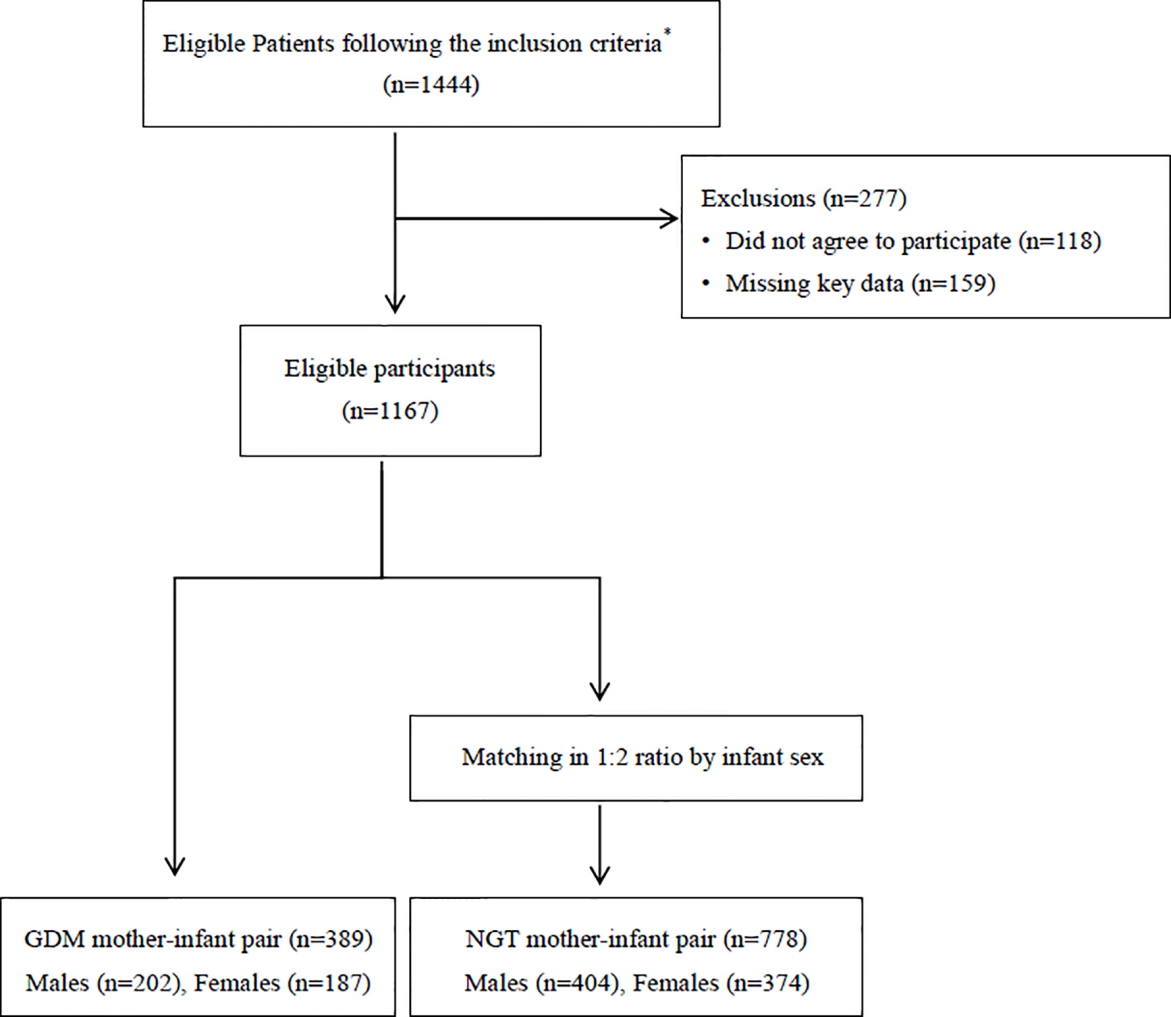
Flow diagram summarizing the inclusion of patients with gestational diabetes in the study.*The inclusion criteria were as follows: 1) Singleton pregnancy; 2) Chinese Han origin; 3) Completion of a 2 hours, 75-g oral glucose tolerance test (OGTT) at 24–28 gestational weeks; 4) No history of a pre-pregnancy or early pregnancy abnormal glucose tolerance; 5) Maternal age between 18 and 45 years; 7) Live infant, and 9) Access complete medical history; GDM, gestational diabetes mellitus; NGT, normal glucose tolerance.

Maternal GDM cases were matched with non GDM cases according to offspring’s gender in a ratio of 1:2, thus the final study group consisted of 1167 mother-infant pairs including 778 NGT pregnancies which included 404 male infants and 374 female infants, and 389 GDM pregnancies which included 202 male infants and 187 female infants. **Figure 1** depicts the study flow chart. The study was approved by the research ethics board of Ruijin Hospital, School of Medicine, Shanghai Jiao Tong University.

### Data Collection

Maternal demographics including age, gravidity and parity, gestational age, presence of gestational hypertension or preeclampsia, insulin use during pregnancy, OGTT values, family history of DM, and pre-pregnancy BMI were extracted from the hospital’s medical records as were delivery data (mode of delivery, newborn sex, birth weight, birth length, and gestational age at delivery). These maternal data was obtained between January 2016 and December 2017 when this study started and infant feeding status and anthropometric data at 1 year of age were prospectively and consecutively collected between January 2018 and December 2019.

Infant feeding status, anthropometric measures at 1 year of age, and parental tobacco consumption were collected using a structured questionnaire when infants were 13 months to 17 months old. The second part of the data collection included a follow-up telephone interview. This was a structured conversation, which included an introduction and obtainment of an informed consent. There is a routine follow-up for every mother when the infant reaches 12 months old in Shanghai, normally completed at the local mother-child clinic in the district. Trained nurses measure the infant’s length (to the nearest 0.1 cm) and weight (to the nearest 0.1 kg) and give written records to the mothers. Questionnaire data were based on these written medical records as reported by the mother. Offspring BMI was calculated accordingly (weight in kg/recumbent length, in meter, squared). Breastfeeding represents mothers who have used breastfeeding and the breastfeeding ratio accounts for more than 50% of the infant’s daily intake. Completed months represents the length of breastfeeding month. For example, when the number of completed months is 6, it means that the infant was breastfed within the first 6 months after birth, and received totally formula milk after 6 months till weaning. The de-identified data is saved in the office in the department of Obstetrics and Gynecology, Ruijin Hospital, Shanghai Jiaotong University School of Medicine, and available for researchers to access.

### Gestational Diabetes Mellitus

We used one-step 2-h 75g OGTT test to screen GDM at 24–28 gestation weeks, women were classified as having GDM in the index pregnancy if any abnormal plasma glucose values were obtained. Abnormal values were defined according to the following standard diagnostic criteria established by the International Association of Diabetes and Pregnancy Study Group: a fasting level of 92 mg/dl (5.1 mmol/liter) or greater, a 1-h value of 180 mg/dl (10.0mmol/liter) or greater, and a 2-h value of 153 mg/dl (8.5mmol/liter) or greater.

### Outcomes

The primary outcome was offspring overweight or obesity status at 1 year of age. Overweight and obesity were defined according to the WHO Child Growth Standards from 2007 based on the length-based BMI-for-age. Overweight was considered as a BMI above the 85^th^ percentiles (≥18.3 kg/m^2^ for a male infant and ≥17.9 kg/m^2^ for a female infant), whereas obesity was considered as a BMI of above the 95^th^ percentile (≥19.2 kg/m^2^ for a male infant and ≥19.0 kg/m^2^ for a female infant). The intra- and inter-assay coefficients of variation were in the range of 9.9% to 10.4%

### Statistical Analysis

Data are expressed as mean ± SD or counts with proportions. Between group differences were compared using the Student *t* test and the Chi-square test/Fisher exact tests, as appropriate. Multivariable logistic regression analysis was used to obtain adjusted odds ratios (ORs) with 95% confidence intervals (CIs).

The co-variables considered for the regression model included maternal gestational diabetes (dichotomic), presence of gestational hypertension or preeclampsia (dichotomic), maternal age (dichotomic over or under 35 years), insulin use during pregnancy (dichotomic), family history of diabetes (dichotomic), parity (dichotomic according to primiparity status), pre-pregnancy BMI (kg/m^2^), gestational weight gain (kg), parental smoking (dichotomic), mode of delivery (vaginal, cesarean), offspring sex (male/female), gestational age upon delivery (weeks), birth weight (kg), breastfeeding status (dichotomic), and weaning months. The study sample size calculation was performed using the WinPepi program. In order to detect an effect of 2% on the median BMI difference for males and females respectively, with an 85% power, and a 5% alpha error rate, a sample size of 674 cases in the NGT group and of 337 in the GDM group was needed. In the presence of a significant interaction (*p* < 0.05), the adjusted odds ratios in separate strata (e.g. male, female) were presented. The sample size was sufficient for a multivariate regression model analysis (20).

The selection of covariates is based on the relevant factors suggested in previous studies that may have an impact on offspring obesity. We used forced entry of potential confounders. The collinearity diagnosis of all variables showed that the variance inflation factor of all variables is less than 5 and the collinearity tolerance is greater than 0.1.

Data were analyzed using SPSS statistics v22.0 (IBM Corp, Los Angeles, CA, USA). A *p* value of <0.05 was considered significant.

## Results

As shown in **Table 1**, pregnant women with GDM were older than controls, with a higher proportion of elderly gravidas but with lower rates of primiparity. There was no significant difference between the groups in rates of family history of diabetes. The GDM group showed a higher mean pre-pregnancy BMI. Chronic hypertension was roughly three-fold higher in the GDM group and preeclampsia was twice as common. The proportion of premature delivery (before 37 week) was higher in the GDM group as was the cesarean delivery rate. Gestational weight gain, newborn sex, birth weight, and birth length were comparable between the groups. Fewer mothers in the GDM group breastfed their child as compared to controls, but overall breastfeeding rates were high. All women in this study denied tobacco use, and the proportions of paternal and family members’ tobacco use were comparable between groups. There was a significant interaction in the association between gestational diabetes and total infant overweight or obesity (BMI≥85th percentile) between males and females (*p* < 0.01).

**Table 1:** Characteristics of the mothers and their offspring between normal glucose tolerance and GDM.

	GDM (n = 389)	NGT (n = 778)	*P* value
**Mothers**			
Age	32.1 ± 4.2	30.0 ± 3.9	<0.01
Age>35 y (%)	122 (31.4)	103 (13.2)	<0.001
Family history (GDM or DM)	91 (23.3)	148 (19.0)	0.08
Primiparous	281 (72.2)	630 (80.9)	<0.001
Pre-pregnancy BMI	28.4 ± 3.9	26.6 ± 3.0	<0.01
Gestational weight gain	12.4 ± 4.5	13.7 ± 3.9	0.24
Hypertension			
Chronic hypertension	31 (7.8)	22 (2.8)	<0.001
Gestational hypertension	34 (8.6)	48 (6.1)	0.10
Preeclampsia	22 (5.5)	17 (2.1)	<0.01
**Newborns**			
Cesarean delivery	249 (64.2)	372 (47.8)	<0.001
Sex (male)	202 (51.9)	404 (51.9)	0.49
Birth weight (g)	3347 ± 534	3322 ± 397	0.23
Birth length (cm)	49.5 ± 1.7	49.6 ± 1.1	0.27
Gestational age (week)	38.2 ± 1.6	38.8 ± 1.2	<0.01
Premature delivery (before 37 wk)	32 (8.2)	33(4.2)	<0.01
**Feeding status and tobacco use**			
Breastfeed (%)	340 (87.4)	727 (93.4)	<0.001
-completed months	6.7 ± 2.4	6.8 ± 2.4	0.21
Exclusively breastfeed (%)	37 (9.5)	86 (11.0)	0.41
Weaning months	10.8 ± 2.0	10.9 ± 2.0	0.83
Parental tobacco use	92 (23.6)	170 (21.8)	0.48
Family member tobacco use	177 (45.5)	359 (46.1)	0.83

Data are given as n (%) or mean ± SD. GDM, gestational diabetes mellitus; NGT, normal glucose tolerance; DM, diabetes mellitus; BMI, body mass index.

Small-for-gestational-age (SGA) and large-for-gestational-age (LGA) were defined by birth weights below the 10th or above the 90th percentile, respectively according to the Chinese neonatal birth weight curve (21). For SGA, there were 19 (4.7%) males in NGT and 10 (4.9%) males in GDM group whereas 18 (4.8%) females in NGT group and 12 (6.4%) females in GDM group. There was no difference founded between these two groups for SGA ratio. For LGA, there were 39 (9.1%) males in NGT and 26 (12.8%) males in GDM group whereas 24 (6.1%) females in NGT group and 16 (8.5%) females in GDM group. The LGA ratio in GDM groups was higher than in NGT group in both sex (*p* < 0.05). The percentage of fetal macrosomia was similarly higher in GDM group (11.8% in total) than in NGT group (7.9% in total) (*p* < 0.05).

**Table 2** presents the anthropometry outcomes at one year of age in offspring of both groups. There was no significant difference in the overall rate of overweight and obesity between the groups. Compared with the female offspring of mothers without GDM, the 1-year-old female offspring of mothers with GDM exhibited a higher mean BMI among females, as well as a higher rate of overweight or obesity. Obesity alone was twice as common in exposed female offspring. In males however, no significant difference was noted in mean BMI and in obesity rates between the groups, and the rate of overweight or obesity (combined) was significantly lower in the GDM group.

**Table 2:** Outcomes at 1 year of age in offspring of mothers with normal glucose tolerance and mothers with GDM.

	Mothers with GDM(total n = 389)	Mothers with NGT(total n = 778)^a^	*P* value
**Anthropometry**			
BMI (kg/m^2^)			
-Overall	16.7 ± 1.6	16.6 ± 1.6	0.19
-Male	16.3 ± 1.5	16.5 ± 1.6	0.08
-Female	17.2 ± 1.8	16.6 ± 1.6	< 0.01
Overweight or obesity (BMI≥85th percentile)			
-Overall (%)	76 (19.5)	138 (17.7)	0.4
-Male (%)	14 (6.9)	50 (12.3)	0.03
-Female (%)	62 (33.1)	88 (23.5)	0.01
Obesity (BMI≥95th percentile)			
-Overall (%)	28 (7.1)	43(5.5)	0.26
-Male (%)	2 (0.9)	14 (3.4)	0.07
-Female (%)	26 (13.9)	29 (7.7)	0.02

a Data are given as n (%) or mean ± SD. 404males in NGT group and 202 in GDM group whereas 374 females in NGT group and 187 in GDM group. GDM, gestational diabetes mellitus; NGT, normal glucose tolerance; BMI, body mass index.

**Table 3** depicts the unadjusted and adjusted odds ratios for obesity or overweight in 1-year old offspring stratified by maternal GDM. Maternal GDM was found to be significantly associated with female offspring overweight or obesity (OR 1.61, 95%CI 1.09–2.37, *p* < 0.01). An opposite association was found in male offspring (OR 0.52, 95%CI 0.28–0.79, *p* < 0.01). These associations remained significant, *in females only*, after adjustments for maternal age, family history of diabetes, parity, pre-pregnancy BMI, insulin use, maternal gestational hypertension, gestational age, birth weight, birth length, delivery mode, paternal smoking, breastfeeding, and weaning months.

**Table 3:** Multivariable models for the association of GDM and offspring overweight or obesity (BMI≥85th percentile) at 1 year of age.

	Unadjusted	Model1	Model2	Model3
ORs^a^				
Total				
-NGT	*Ref*	*Ref*	*Ref*	*Ref*
-GDM	1.23 (0.82, 1.53)	1.01 (0.72, 1.41)	1.04 (0.74, 1.45)	1.07 (0.76, 1.50)
Male				
-NGT	*Ref*	*Ref*	*Ref*	*Ref*
-GDM	0.52 (0.28, 0.97)^*^	0.52 (0.27, 1.02)	0.53 (0.27, 1.02)	0.51 (0.26, 1.01)
Female				
-NGT	*Ref*	*Ref*	*Ref*	*Ref*
-GDM	1.61 (1.09, 2.37)^*^	1.42 (0.86, 2.15)	1.47 (0.96, 2.25)	1.60 (1.03, 2.47)^*^

a Data presented are the odds ratios (95% CI).404 males in NGT group and 202 in GDM group whereas 374 females in NGT group and 187 in GDM group.

Model 1 adjusted for maternal age, family history of diabetes, parity, gestational weight gain, Pre-pregnancy BMI, and maternal gestational hypertension.

Model 2 adjusted for the same variables as model 1, plus gestational age, birth weight, birth length, and mode of delivery.

Model 3 adjusted for the same variables as model 2, plus parental smoking, breastfeeding status, and weaning months.

GDM, gestational diabetes mellitus; NGT, normal glucose tolerance; BMI, body mass index.

^*^p <0.05.

**Table 4** presents the adjusted OR of the association between breastfeeding status and infant overweight or obesity. The model reflects that breastfeeding may have a protective effect against overweight or obesity in female offspring at 1 year of age.

**Table 4:** The association between breastfeed and infant overweight or obesity (BMI≥85th percentile).

			Total			Male				Female	
aORs^a^		NB	Breastfeed	*P* value	NB	Breastfeed	*P* value		NB	Breastfeed	*P* value
		*Ref*	0.24 (0.09, 0.61)	<0.01	*Ref*	0.24 (0.05,1.05)	0.059		*Ref*	0.22 (0.06, 0.79)	0.02

a Data presented are the adjusted odds ratios (95% CI). Total n=1167, whereas 1067 in Breastfeed group and 100 in NB group; Male n=606, whereas 542 in Breastfeed group and 64 in NB group; Female n=561, whereas 525 in Breastfeed group and 36 in NB group. Odds ratios were adjusted for gestational diabetes, maternal age, family history of diabetes, parity, gestational weight gain, Pre-pregnancy BMI, maternal gestational hypertension, gestational age, birth weight, birth length, mode of delivery, and parental smoking.

GDM, gestational diabetes mellitus; NGT, normal glucose tolerance; BMI, body mass index; NB, not breastfeed.

The **Supplementary Tables (1–3)** repeat the same analyses as previously described, but with infant obesity only (BMI≥95th percentile) as the studied outcome. The association between GDM and infant obesity only, in females, remained significant (**Supplementary Table 2**) while adjusting for multiple confounders.

## Discussion

### Main Findings

In this prospective cohort study, we found GDM to be independently associated with increased overweight or obesity risk in female offspring at age 1 year but not in male offspring. Our results showed a gender related difference in the association of GDM and infant obesity status in the early postnatal period.

### Data Interpretation and Comparisons With Findings in Previous Studies

Many previous studies focused on the associations between maternal GDM status and the risk of childhood overweight and obesity. Studies of the Northwestern Diabetes in Pregnancy Study in Chicago, on Pima Indian (13), the HAPO study in Hongkong (22), a large nationwide cohort study conducted in the United States (8), and Kaiser Permanente centers study (7) claimed to have found a significant positive association between maternal GDM and childhood obesity, while other studies (including the HAPO study in the UK) did not found a significant result (23, 24). Most studies set the ending point at age 5 years or older, which may be effected by potential confounders at this rapid growth phase (16, 25–27). The outcomes from The National Collaborative Perinatal Project indicated the positive association between GDM and childhood overweight at age 4 and 7 but not at age 3 years of age (28). Pham et al. reported the negative association between GDM and childhood obesity in children aged 2–4 years. Inconsistent results could be caused by the distinct time-point of infant measure and the difference in gender (19). In the study from Tam et al, the difference of overweight ratio in offspring between maternal GDM and non GDM only found in female at 7 years of age which is similar to our result (22). In the present study, we observed a graded effect of GDM on the offspring’s risk of overweight or obesity at 1 year of age in female offspring may indicate a gender specific effect of GDM in determining the metabolic status of an infant during the first year of life.

In agreement with previous studies, breastfeeding was found to have a protective effect in offspring against childhood obesity and overweight (29, 30). In this study, we found the breastfeeding rate in NGT female offspring to be significantly higher than in GDM female offspring (as shown in **Supplementary Table 1**), whereas no significant difference was found in breastfeeding rates between NGT and GDM males. Regression model 3 presented in **Table 3**, proves the association between maternal GDM and female offspring overweight and obesity to be independent of breastfeeding status. Future study may be needed to rule out confounding factors due to differences in breastfeeding status.

The sex-specific effects of GDM on body composition of school age child were reported by Regnault et al, they indicated the positive association between GDM and increased overall adiposity only in male offspring (31). One animal study have reported that maternal fructose intake during gestation can lead to sex-specific changes in fetuses where only female neonatal showed increased blood glucose and hyperleptinemia, but reduced β-hydroxybutyrate, which may caused by alteration in placental growth (32). The gender related difference in the association of GDM and infant obesity status may caused by different maternal eating habits which reflected by higher pre-pregnancy BMI, however we did not find a significant difference in pre-pregnancy BMI between women carrying male or female offspring in both GDM and NGT groups. Further studies are needed to investigate the underlying mechanism of the sex specific effect.

### Strengths and Limitations

Strengths of the study include the high rates of infant follow up, and the analyses accounting for prenatal and postnatal potential confounding factors. This study is observational in nature. Our findings suggest significant association but not causation or underlying pathophysiology. The data was obtained from a single center, thus generalizability necessitates confirmation. In addition, some of the data was self-reported by the mothers.

## Conclusion

Maternal GDM is significantly associated with increased overweight or obesity risk in female offspring at 1 year of age, and this effect appears to be sex specific.

## Data Availability Statement

The raw data supporting the conclusions of this article will be made available by the authors, without undue reservation.

## Ethics Statement

The studies involving human participants were reviewed and approved by The research ethics board of Ruijin Hospital Research Center, Shanghai Jiaotong University. The patients/participants provided their written informed consent to participate in this study.

## Author Contributions

QD, AW, YS and WF developed the research protocol and obtained the research grants. QD, WF, HZ, and YL contributed to the acquisition of research data. QD contributed to the data analyses. QD drafted the manuscript. All authors contributed to the article and approved the submitted version.

## Funding

This work is supported by Shanghai Science and Technology Committee (grant# 17411950504), Shanghai Sailing Program (grant# 18YF1414400) and National Natural Science Foundation of China (grant# 81901488). The funders had no role in study design, data collection, analysis, interpretation, or writing of the report.

## Conflict of Interest

The authors declare that the research was conducted in the absence of any commercial or financial relationships that could be construed as a potential conflict of interest.

## Abbreviations

GDM, gestational diabetes mellitus; NGT, normal glucose tolerance; OGTT, oral glucose tolerance test; DM, diabetes mellitus.
