# Supplementary material for: The Sex Specific Association Between Maternal Gestational Diabetes and Offspring Metabolic Status at 1 Year of Age
Source: Front Endocrinol (Lausanne). 2021 Feb 9;11:608125. doi: 10.3389/fendo.2020.608125 (PMC7900617; doi:10.3389/fendo.2020.608125)
Supplement: Supplementary file 1 [file Table_1.docx]

**Supplementary Table1.** Characteristics of the mothers and their offspring stratified by glucose tolerance and sex.

|  | **GDM** | **NGT** | ***P* value** |
| --- | --- | --- | --- |
| **Feeding status and tobacco use** |  |  |  |
| **Males** | **n=202** | **n=404** |  |
| Breastfeed (%) | 178 (88.1) | 364 (90.0) | 0.45 |
| -completed months | 6.7 ± 2.4 | 6.8 ± 2.3 | 0.21 |
| Exclusively breastfeed (%) | 22 (10.8) | 47 (11.6) | 0.78 |
| -completed months | 12.4 ± 1.7 | 11.8 ± 1.9 | 0.88 |
| Weaning months | 11.0 ± 2.1 | 10.9 ± 2.0 | 0.81 |
| Parental tobacco use | 50 (24.7) | 93 (23.0) | 0.63 |
| Family member tobacco use | 93 (46.0) | 198 (49.0) | 0.49 |
| **Females** | **n=187** | **n=374** |  |
| Breastfeed (%) | 162 (86.6) | 363 (97.0) | < 0.001 |
| -completed months | 6.8 ± 2.4 | 6.8 ± 2.5 | 0.27 |
| Exclusively breastfeed (%) | 15 (8.0) | 39 (10.4) | 0.36 |
| Weaning months | 10.7 ± 1.7 | 10.9 ± 1.9 | 0.23 |
| Parental tobacco use | 42 (22.4) | 77 (20.5) | 0.60 |
| Family member tobacco use | 84 (44.9) | 161 (43.0) | 0.67 |

Data are given as n (%) or mean ± SD.GDM, gestational diabetes mellitus; NGT, normal glucose tolerance; DM, diabetes mellitus; BMI, body mass index.

**Supplementary Table2.** Multivariable models for the association of GDM and offspring obesity (BMI≥95th percentile) at 1 year of age.

|  | **Unadjusted** | **Model1** | **Model2** | **Model3** |
| --- | --- | --- | --- | --- |
| ORs ^a^ |  |  |  |  |
| Total |  |  |  |  |
| -NGT | *Ref* | *Ref* | *Ref* | *Ref* |
| -GDM | 1.32 (0.81, 2.16) | 1.15 (0.68, 1.96) | 1.19 (0.70, 2.04) | 1.36 (0.78, 2.37) |
| Male |  |  |  |  |
| -NGT | *Ref* | *Ref* | *Ref* | *Ref* |
| -GDM | 0.27 (0.06, 1.23) | 0.23 (0.49, 1.17) | 0.25 (0.52, 1.25) | 0.26 (0.05, 1.33) |
| Female |  |  |  |  |
| -NGT | *Ref* | *Ref* | *Ref* | *Ref* |
| -GDM | 1.92 (1.09, 3.36) ^*^ | 1.60 (0.86, 2.96) | 1.69 (0.91, 3.14) | 2.04 (1.07, 3.85) ^*^ |

GDM, gestational diabetes mellitus; NGT, normal glucose tolerance; BMI, body mass index.

^a^ Data presented are the odds ratios (95% CI).

404 males in NGT group and 202 in GDM group whereas 374 females in NGT group and 187 in GDM group.

Model 1 adjusted for maternal age, family history of diabetes, parity, gestational weight gain, Pre-pregnancy BMI, and maternal gestational hypertension.

Mosel 2 adjusted for the same variables as model 1,plus gestational age, birth weight, birth length, and mode of delivery.

Model 3 adjusted for the same variables as model 2, plus parental smoking, breastfeeding status, and weaning months.

^*^ p <0.05.

**Supplementary Table3.** The association between breastfeeding status and infant obesity (BMI≥95th percentile)

|  |  | **Total** |  |  | **Male** |  |  |  | **Female** |  |
| --- | --- | --- | --- | --- | --- | --- | --- | --- | --- | --- |
| aORs ^a^ | NB | Breastfeed | *P* value | NB | Breastfeed | *P* value |  | NB | Breastfeed | *P* value |
|  | *Ref* | 0.57 (0.17, 1.87) | 0.35 | *Ref* | 1.28 (0.26,6.08) | 0.75 |  | *Ref* | 0.24 (0.03, 1. 93) | 0.18 |

^a^ Data presented are the adjusted odds ratios (95% CI). Total n=1167, whereas 1067 in Breastfeed group and 100 in NB group; Male n=606, whereas 542 in Breastfeed group and 64 in NB group; Female n=561, whereas 525 in Breastfeed group and 36 in NB group. Odds ratios were adjusted for gestational diabetes, maternal age, family history of diabetes, parity, gestational weight gain, Pre-pregnancy BMI, maternal gestational hypertension, gestational age, birth weight, birth length, mode of delivery, and parental smoking.

GDM, gestational diabetes mellitus; NGT, normal glucose tolerance; BMI, body mass index; NB, not breastfeed.
